# Discovery of α-Linolenic Acid 16(*S*)-Lipoxygenase: Cucumber (*Cucumis sativus* L.) Vegetative Lipoxygenase 3

**DOI:** 10.3390/ijms241612977

**Published:** 2023-08-19

**Authors:** Svetlana S. Gorina, Alevtina M. Egorova, Natalia V. Lantsova, Yana Y. Toporkova, Alexander N. Grechkin

**Affiliations:** Kazan Institute of Biochemistry and Biophysics, FRC Kazan Scientific Center of RAS, P.O. Box 261, 420111 Kazan, Russia; egorova@kibb.knc.ru (A.M.E.); natamed2@yandex.ru (N.V.L.); toporkova@kibb.knc.ru (Y.Y.T.)

**Keywords:** lipoxygenase, molecular cloning, ω3 fatty acids, ω3(*S*) dioxygenation, fatty acid hydroperoxides, cucumber (*Cucumis sativus* L.)

## Abstract

The GC-MS profiling of the endogenous oxylipins (Me/TMS) from cucumber (*Cucumis sativus* L.) leaves, flowers, and fruit peels revealed a remarkable abundance of 16-hydroxy-9,12,14-octadecatrienoic acid (16-HOT). Incubations of homogenates from these organs with α-linolenic acid yielded 16(*S*)-hydroperoxide (16-HPOT) as a predominant product. Targeted proteomic analyses of these tissues revealed the presence of several highly homologous isoforms of the putative “9*S*-lipoxygenase type 6”. One of these isoenzymes (CsLOX3, an 877 amino acid polypeptide) was prepared by heterologous expression in *E. coli* and exhibited 16(*S*)- and 13(*S*)-lipoxygenase activity toward α-linolenic and linoleic acids, respectively. Furthermore, α-linolenate was a preferred substrate. The molecular structures of 16(*S*)-HOT and 16(*S*)-HPOT (Me or Me/TMS) were unequivocally confirmed by the mass spectral data, ^1^H-NMR, 2D ^1^H-^1^H-COSY, TOCSY, HMBC, and HSQC spectra, as well as enantiomeric HPLC analyses. Thus, the vegetative CsLOX3, biosynthesizing 16(*S*)-HPOT, is the first 16(*S*)-LOX and ω3-LOX ever discovered. Eicosapentaenoic and hexadecatrienoic acids were also specifically transformed to the corresponding ω3(*S*)-hydroperoxides by CsLOX3.

## 1. Introduction

Lipoxygenase (LOX—linoleate:oxygen oxidoreductase; EC 1.13.11.12) is a widespread enzyme in aerobic organisms [1,2,3]. These enzymes convert the methylene-interrupted polyenoic fatty acids to cytotoxic hydroperoxides. As a rule, LOXs exhibit a high degree of stereospecificity and regiospecificity. LOXs are the key enzymes controlling the pathways of biosynthesis of numerous bioactive oxylipins, including leukotrienes, lipoxins, protectins, and resolvins, in mammals [4,5], as well as jasmonates and their precursors, aldehydes, divinyl ethers, epoxyalcohols, and trihydroxyacids in plants [1,3].

While both human [5] and Arabidopsis [6] genomes include 6 LOX genes, the soybean (*Glycine max*) genome includes at least 37 expressed LOX genes deposited in the NCBI database. The specificity and role of many plant LOX isoenzymes are still not fully understood. Large parts of the LOX isoenzymes (even the soybean LOXs, which are the classical models) known from genomic data have not been studied as recombinant proteins.

Regio- and stereospecificities of fatty acid dioxygenation largely establish the directions of conversions of fatty acid hydroperoxides to various oxylipins. Plant LOXs are well known to oxidize linoleic and α-linolenic acids to 13(*S*) and 9(*S*)-hydroperoxides. α-Linolenic acid can theoretically be oxidized to its 12- and 16-hydroperoxides (12-HPOT and 16-HPOT, respectively). These hydroperoxides, as well as the related 12- and 16-hydroxy derivatives (12-HOT and 16-HOT, respectively), have so far been considered mainly to be the products of α-linolenate autoxidation [7,8]. There are some reports on the detection of enantiopure 12(*R*)-HOT [9,10], dinor-12(*R*)-HOT [11], and 12(*S*)-HOT [12,13,14], as well as 16(*S*)-HOT [15] or 16(*R*)-HOT [12,13], in plants and algae. However, their biosynthetic origin has not been uncovered yet. To our knowledge, there are only a few examples of LOX oxidation of α-linolenic acid to 12-HPOT. Firstly, the recombinant LOX domain of the chimeric catalase/LOX protein of the blue-green alga *Acaryochloris marina* oxidizes α-linolenic acid to 12(*R*)-HPOT [16]. The same specificity was found for LOX of the blue-green alga *Cyanothece* sp. [17]. Furthermore, 12(*S*)-LOX was recently detected in the proteobacterium *Myxococcus xanthus* [18].

When studying the oxylipin profiles of cucumber plants, we unexpectedly observed a bulk abundance of 16-HOT in the leaves, fruit peels, and flowers. Further proteomic analyses allowed us to identify a few highly homologous LOX isoforms present in these tissues. Molecular cloning of one of them resulted in the preparation of the recombinant protein CsLOX3, which utilized α-linolenic acid as a preferred substrate and possessed unprecedented 16(*S*)-LOX activity. The obtained results are reported in the present work.

## 2. Results

### 2.1. Hydro(pero)xy Fatty Acid Patterns of Cucumber Seedlings and Fruits—Detection of 16(S)-HPOT

**Figure 1:**
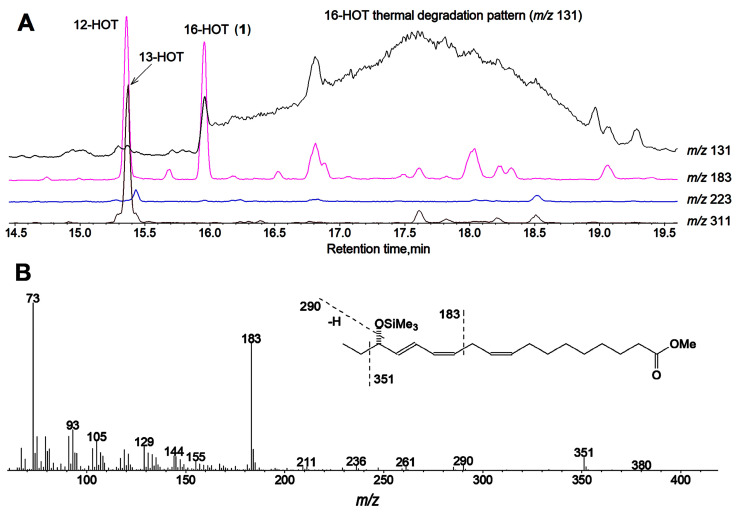
GC-MS analyses of NaBH_4_-reduced products (Me/TMS) of α-linolenic acid oxidation by LOX preparation from cucumber flowers. (**A**) The selected ion GC-MS chromatograms at *m*/*z* 131, 183, 223, and 311. (**B**) Mass spectrum for product **1**; inset, mass fragmentation scheme.

Our preliminary data of GC-MS profiling of oxylipins (Me/TMS) of cucumber flowers, leaves, and fruit peels revealed the presence of prominent oxylipin **1** besides 13-HOT, 12-HOT, and 9-HOD. For the identification of product **1**, in vitro incubations of α-linolenic acid with cell-free preparations from cucumber flowers or fruit peels were performed. The GC-MS analysis data of NaBH_4_-reduced products (Me/TMS) of α-linolenic acid incubation with flower preparation are presented in Figure 1. The mass spectrum (Figure 1B) of product **1** (Me/TMS) possessed M^+^ at *m*/*z* 380 (0.4%), [M − Et]^+^ at *m*/*z* 351 (6%), [M − TMSOH]^+^ at *m*/*z* 290 (3%), [351 − TMSOH]^+^ at *m*/*z* 261 (2%), *m*/*z* 236 (3%), [M − C1/C11]^+^ at *m*/*z* 183 (63%), *m*/*z* 129 (13%), *m*/*z* 105 (20%), *m*/*z* 91 (27%), *m*/*z* 75 (33%), and [SiMe_3_]^+^ at *m*/*z* 73 (100%). Its fragmentation patterns (Figure 1B, inset) advocated the structure of 16-HOT. One should note that the GC-MS analyses of 16-HOT (Me/TMS) were accompanied by thermal rearrangement, resulting in a wide and intensive tailing pattern of the 16-HOT peak. This trace exhibited a distinct mass spectrum, which possessed a base peak [EtCH=O^+^ − SiMe_3_] at *m*/*z* 131. Cleavage between C15 and C16 indicated the tertiary structure of C15, presumably due to the thermal 1,3-butadiene-cyclobutene rearrangement [19,20] of conjugated double bonds. This thermal conversion also occurs with different hydroxy fatty acids, like 13-HOD or 13-HOT (Me/TMS), but it is significantly less abundant than in the case of 16-HOT (Me/TMS). The abundant thermal degradation of 5-HETE (Me/TMS) was also observed previously [21].

The catalytic hydrogenation over PtO_2_ converted compound **1** to a saturated analog, the mass spectrum of which possessed [M − H]^+^ at *m*/*z* 385 (0.4%), [M − Me]^+^ at *m*/*z* 371 (0.6%), [M − Et]^+^ at *m*/*z* 357 (23%), *m*/*z* 339 (9%), [M − C16/C18 + TMS]^+^ at *m*/*z* 328 (6%), *m*/*z* 159 (8%), *m*/*z* 146 (6%), [M − C1/C15]^+^ at *m*/*z* 131 (100%), *m*/*z* 97 (10%), *m*/*z* 75 (20%), and [SiMe_3_]^+^ at *m*/*z* 73 (47%). These data suggest the structure of 16-hydroxystearic acid (Me/TMS) for hydrogenated products, thus confirming the structure of 16-HOT for compound **1**. For further approval of identification, the products (Me esters) of α-linolenate oxidation by enzyme preparation from cucumber flowers were sequentially separated and purified by micropreparative RP-HPLC and NP-HPLC.

**Figure 2:**
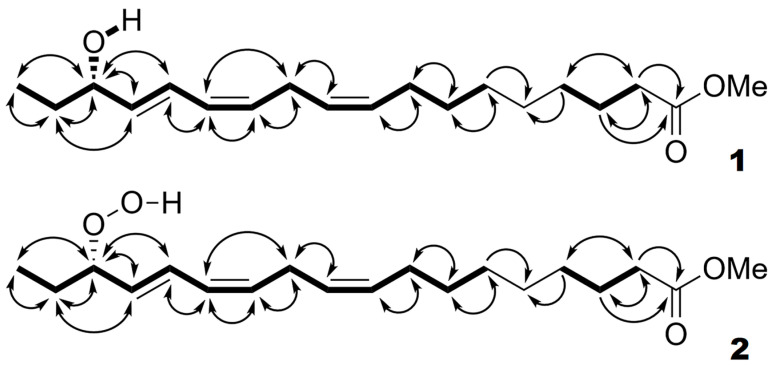
The observed ^1^H-^1^H-COSY (bold lines) and ^1^H-^13^C-HMBC (curved arrows) correlations of compounds **1** and **2**.

**Figure 3:**
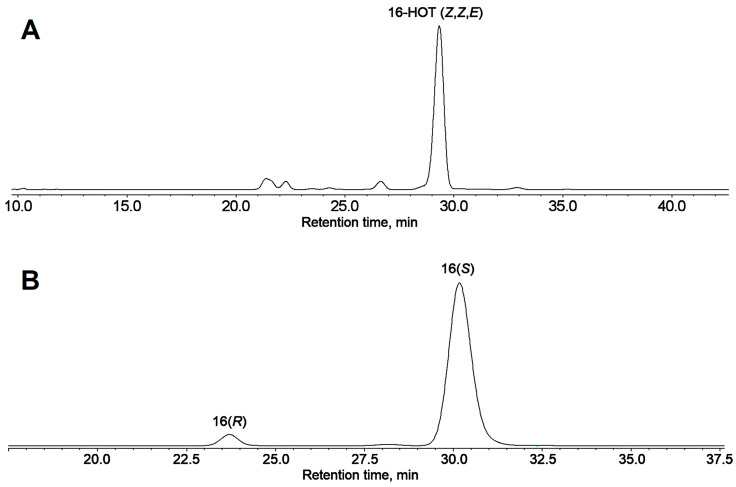
Normal phase HPLC (NP-HPLC) and chiral phase HPLC (CP-HPLC) analyses of NaBH_4_-reduced products (Me esters) of α-linolenic acid incubation with LOX preparation from cucumber flowers. (**A**) The NP-HPLC chromatogram of total NaBH_4_-reduced products (Me). (**B**) the CP-HPLC chromatogram of 16-HOT fraction collected from NP-HPLC column. The ultraviolet absorption (190–370 nm) was monitored by diode array detector, 235 nm chromatograms are shown. Full details of analyses are described in Section 4.

The native predominant product **2** (Me ester) of α-linolenic acid oxidation in the presence of enzyme preparation from flowers was sequentially purified by RP- and NP-HPLC and subjected to NMR spectral records. The NMR spectral data (^1^H-NMR, ^1^H-^1^H-COSY, ^1^H-^1^H-TOCSY, ^1^H-^13^C-HSQC, and ^1^H-^13^C-HMBC, [^2^H_6_]benzene, 303 K) for compound **2** (Me) are presented in Table 1. All signal attributions were substantiated by the ^1^H-^1^H-COSY and ^1^H-^13^C-HMBC correlations (Figure 2), as well as the ^1^H-^1^H-TOCSY correlations (Table 1). The spectrum possessed a signal of hydroperoxyl-bearing methine H16 at 4.24 ppm. Its attribution to H16 was unambiguously confirmed by ^1^H-^1^H-COSY cross-correlations H18/H17 and H17/H16. The data also confirmed the presence of the 9*Z*,12*Z*,14*E* olefinic moiety (Table 1). Overall, the data allow ascribing a structure of 16(*S*)-HPOT, (9*Z*,12*Z*,14*E*,16*S*)-16-hydroperoxy-9,12,14-octadecatrienoic acid (Me) to compound **2** (Me ester). The stereochemical assignment is based on the data for the reduced analog, 16-HOT (as described below, Figure 3B).

The NaBH_4_ reduction converted 16-HPOT (**2**) to product **1**, which was purified (as a Me ester) using NP-HPLC, and then, its NMR spectra (Table 2) were recorded. The spectrum was largely similar to that of compound **2**. The signal of hydroxyl-bearing methine H16 appeared at 3.88 ppm (compared to 4.24 ppm in the case of compound **2**). Its multiplet was more complex compared to the H16 signal of compound **2** due to the additional spin coupling constant, *J*_16,OH_ = 4.1 Hz. Moreover, the spectrum possessed an additional OH signal at 1.04 ppm (broad doublet). All signal attributions were validated by the ^1^H-^1^H-COSY and ^1^H-^13^C-HMBC correlations (Figure 2), as well as the ^1^H-^1^H-TOCSY correlations (Table 2). Overall, the data confirm the identification of compound **1** as 16-HOT (Me). For steric analyses, product **1** (Me ester) was subjected to chiral phase HPLC separations.

The Me esters of the NaBH_4_-reduced products of α-linolenic acid incubation with LOX preparation from cucumber flowers were first purified using RP-HPLC. The fraction of total hydroxy fatty acids was collected and then subjected to NP-HPLC analyses. The predominant product of α-linolenate conversion was 16-HOT (**1**), as seen in Figure 3A. Purified 16-HOT (Me) was subjected to CP-HPLC analyses. As seen in Figure 3B, the 16-HOT was largely enantiopure and composed mainly (95%) of the 16(*S*)-enantiomer. The rest (5%) was the 16(*R*)-enantiomer. Thus, the above-described structural data and the enantiomeric analyses allow one to identify compounds **1** and **2** as 16(*S*)-HOT and 16(*S*)-HPOT, respectively. The specific biosynthesis of 16(*S*)-HPOT indicated the probable involvement of some special LOX isoform. To identify this hypothetical LOX, the below-described targeted proteomic analyses were performed (the results are described below in Section 2.3).

### 2.2. Specificity of Linoleic Acid Oxidation by LOX from Cucumber Flowers

**Figure 4:**
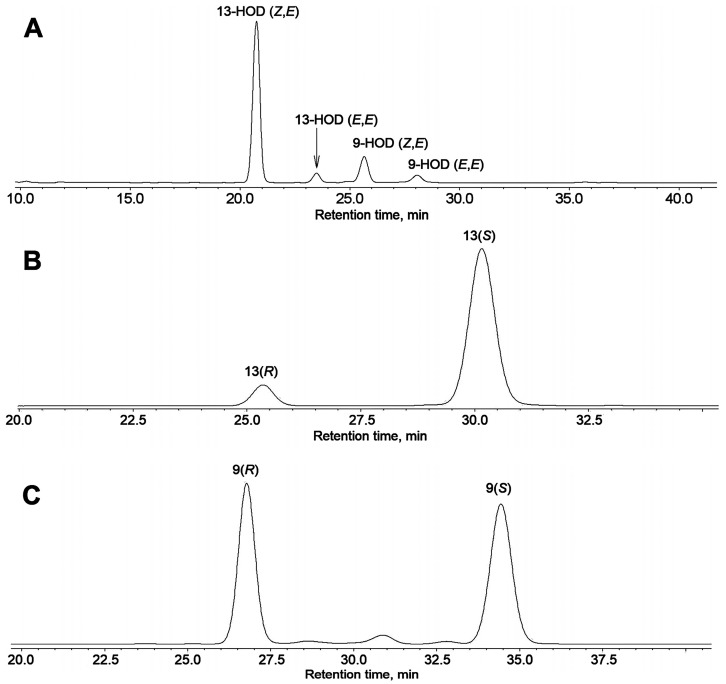
Normal phase HPLC (NP-HPLC) and chiral phase HPLC (CP-HPLC) analyses of NaBH_4_-reduced products (Me esters) of linoleic acid incubation with LOX preparation from cucumber flowers. (**A**) The NP-HPLC chromatogram of total NaBH_4_-reduced products (Me). (**B**) The CP-HPLC chromatogram of 13-HOD (*Z*,*E*) fraction collected from NP-HPLC column. (**C**) The CP-HPLC chromatogram of 9-HOD (*Z*,*E*) fraction collected from NP-HPLC column. The ultraviolet absorption (190–370 nm) was monitored by diode array detector, 235 nm chromatograms are shown. Full details of analyses are described in Section 4.

The in vitro incubations of LOX preparations from cucumber flowers or fruit peels with different polyenoic fatty acids showed that α-linolenic acid was a preferred substrate. Linoleic acid was oxidized with lower efficiency. The NP-HPLC chromatogram of NaBH_4_-reduced products (Me) of linoleic acid oxidation is shown in Figure 4A. Separate peaks were collected and identified by GC-MS analyses as Me/TMS derivatives or totally hydrogenated Me/TMS derivatives. The mass spectral data for linoleic acid oxidation products are not presented. As seen in Figure 4A, linoleic acid was oxidized to (9*Z*,11*E*)-13-hydroperoxide, 13-HPOD, and (10*E*,12*Z*)-9-hydroperoxide, 9-HPOD, at a ratio of ca. 4:1. Moreover, a noticeable yield of (*E*,*E*)-isomers of both 13-HPOD and 9-HPOD was observed.

(9*Z*,11*E*)-13-HPOD and (10*E*,12*Z*)-9-HPOD (Me) were collected after the NP-HPLC separation and subjected to CP-HPLC analyses. The results are presented in Figure 4B,C. As seen in the CP-HPLC data, (9*Z*,11*E*)-13-HPOD was largely (ca. 90%) composed of 13(*S*)-enantiomer and the remainder of 13(*R*)-enantiomer. In contrast, (9*Z*,11*E*)-13-HPOD was largely racemic, composed of 53% of 9(*S*)-enantiomer and 47% of 9(*R*)-enantiomer.

### 2.3. Targeted Proteomic Analyses of LOXs from Cucumber Flowers, Leaves, and Fruits

The polypeptides isolated from the cucumber flowers, as well as the leaves and fruit peel, were separated using 1D SDS-PAGE. The tryptic digestions of the electrophoretic bands corresponding to the expected LOX molecular weight (99 ± 2 kD) were subjected to proteomic analyses. Two LOX isoforms were detected in flowers: proteins NP_001292695.1, identified at 50% sequence coverage, and XP_004150982.1, identified at 27% sequence coverage. In the leaves, there was one LOX isoform, protein KGN60957.2. Similar proteomic analyses of fruit peel uncovered three LOX isoforms: (1) NP_001292695.1, identified at 37% sequence coverage; (2) KGN60957.2, identified at 23% sequence coverage; and (3) XP_031737376.1, identified at 23% sequence coverage. Detailed proteomic data are presented in the Supplementary Information (Supplementary Table S1).

### 2.4. Molecular Cloning of CsLOX3 and Identification of the Recombinant Protein as 16(S)-LOX

Gene GI:101206086 (GenBank, NCBI) encoding protein KGN60957.2 detected in leaves and fruit peel is localized on chromosome 2 (location: 3,050,529..3,054,968) and consists of nine exons. Sequence analysis with publicly available gene localization prediction software revealed that the KGN60957.2 protein lacks a chloroplast transit peptide. This sequence was used for the construction of primers CsLOXF and CsLOXR. The ORF, consisting of 2634 nucleotides and encoding an 877-amino-acid polypeptide, was cloned into the pET-32 Ek/LIC vector, which contained thioredoxin at the N-terminus that increased the soluble fraction of the protein. The resulting polypeptide was 99.77% identical to the GenBank KGN60957.2 protein. Differences were at the following sites: Thr instead of Ala-436 and Lys instead of Asn-712 (Supplementary Figure S1). The recombinant enzyme was referred to as CsLOX3. Recombinant CsLOX3 was obtained in a heterologous expression system using *E. coli* cells and then purified using anion-exchange chromatography. The enzymatic activity was measured by the growth of fatty acid hydroperoxide absorbance at 234 nm. The pH optimum of the recombinant CsLOX3 was 7.5. This pH was used to evaluate the preference of recombinant CsLOX3 toward linoleic or α-linolenic acids. The *K_M_* values (Table 3) demonstrated that recombinant CsLOX3 affinity for α-linolenic acid was higher than for linoleic acid. The estimated turnover rates (*k_cat_*) of linoleic and α-linolenic acid conversions were near equal (Table 3). As judged by specificity constant (*k_cat_*/*K_M_*) values, α-linolenic acid was the preferential substrate for recombinant CsLOX3.

**Figure 5:**
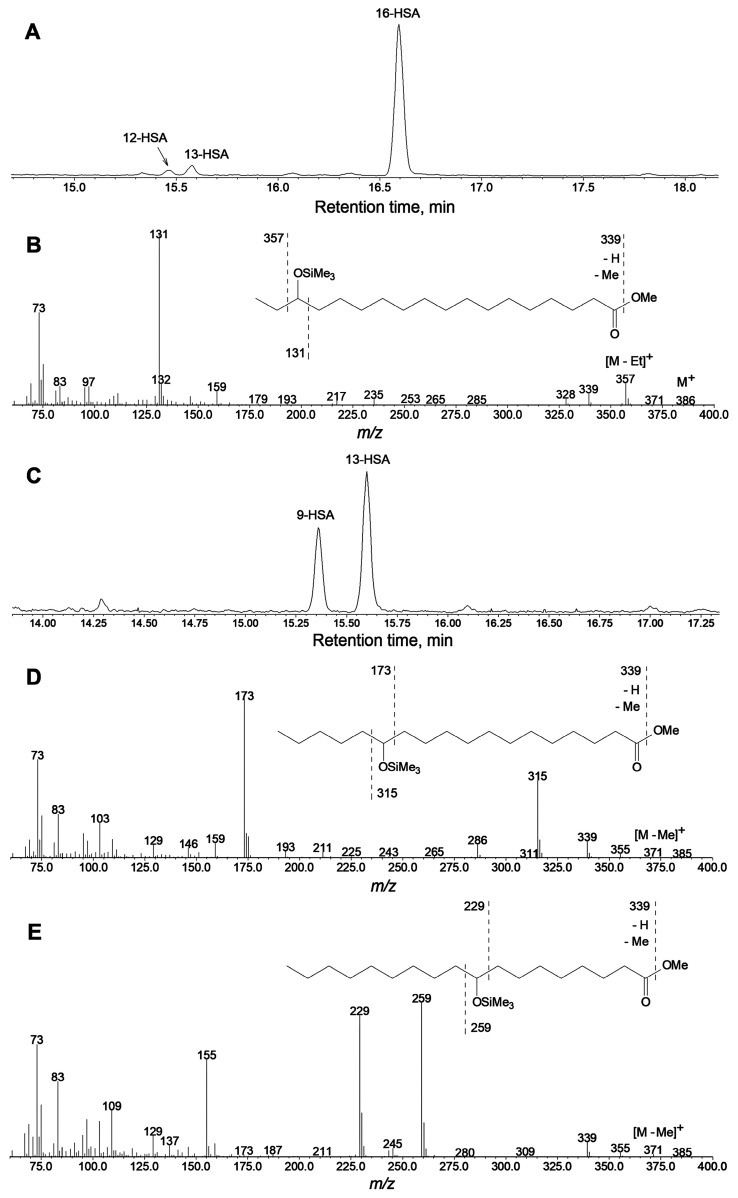
GC-MS total ion current (TIC) chromatograms of products (Me/TMS) of α-linolenic acid (**A**) or linoleic acid (**C**) conversions by the recombinant CsLOX3 after preliminary NaBH_4_ reduction and hydrogenation over PtO_2_; (**B**,**D**,**E**) mass spectra of major (totally reduced and hydrogenated) products (totally reduced and hydrogenated, Me/TMS); insets are the mass fragmentation schemes. (**B**,**D**,**E**) Spectra of 16-HSA, 13-HSA, and 9-HSA and 16-, 13-, and 9-hydroxystearic acid (Me/TMS) formed via the derivatization of 16-HPOT, 13-HPOD, and 9-HPOD, respectively. 16-HSA—16-hydroxystearic acid; 13-HSA—13-hydroxystearic acid; 9-HSA—9-hydroxystearic acid.

The GC-MS analyses of NaBH_4_-reduced products (Me/TMS) showed that CsLOX3 specifically converted α-linolenic acid to product **1**, 16-HOT (Me/TMS). The GC-MS profile of products was analogous to that shown above in Figure 1A. A chromatogram of the products (NaBH_4_-reduced, totally hydrogenated, Me/TMS) of conversion by CsLOX3 is presented in Figure 5A. The predominant product was unequivocally identified by its mass spectrum (Figure 5B) and mass fragmentation patterns (Figure 5B, inset) as 16-hydroxystearic acid (Me/TMS). Thus, the original α-linolenic acid oxidation product was 16-HPOT.

**Figure 6:**
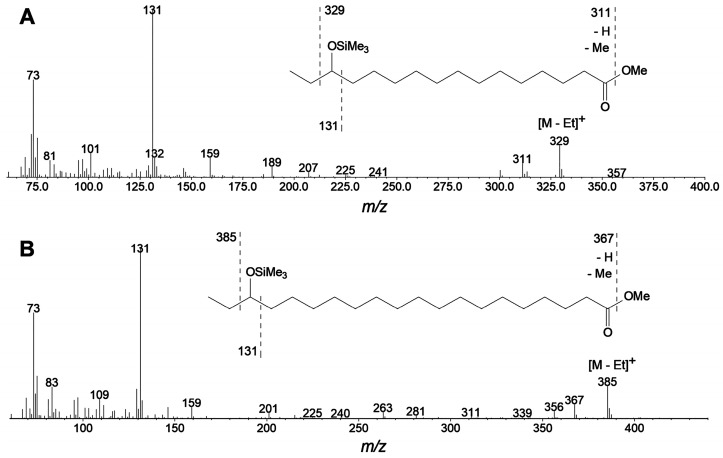
Mass spectra of products (Me/TMS) of (7*Z*,10*Z*,13*Z*)-hexadecatrienoic acid (**A**) or (5*Z*,8*Z*,11*Z*,14*Z*,17*Z*)-eicosapentaenoic acid (**B**) conversions by the recombinant CsLOX3 after preliminary NaBH_4_ reduction and hydrogenation over PtO_2_. Insets (**A**,**B**) are the mass fragmentation schemes for 14-hydroxypalmitic and 18-hydroxyeicosanoic acids (Me/TMS), respectively.

Linoleic acid was a poorer substrate for CsLOX3. It possessed a limited regiospecificity, as seen in the GC-MS chromatogram (Figure 5C) of NaBH_4_-reduced, totally hydrogenated products (Me/TMS). Two isomers, 13-hydroxystearic and 9-hydroxystearic acids (Me/TMS), were detected at a ca. 3:2 ratio (Figure 5C). Their identification was confirmed by mass spectra (Figure 5D,E) and mass fragmentation patterns (insets of the same figures). Furthermore, recombinant CsLOX3 was also tested with (7*Z*,10*Z*,13*Z*)-hexadecatrienoic acid (16:3) and eicosapentaenoic acid (20:5) as the substrates. The oxidation of these two fatty acids occurred specifically (ca. 95% *S*-epimers, and resulted in the formation of ω3-hydro(pero)xy derivatives. The mass spectra of the corresponding products (totally hydrogenated Me/TMS derivatives) are presented in Figure 6A,B. The mass fragmentation patterns (insets of Figure 6A,B) indicated the structures of 14-hydroxypalmitic acid (Me/TMS) and 18-hydroxyeicosanoic acid (Me/TMS), respectively. These data advocated the structures of 14-hydroperoxy-7,10,12-hexadecatrienoic (14-HPHT) and 18-hydroperoxy-5,8,11,14,16-eicosapentaenoic acids (18-HPEPE). Arachidonic acid was oxidized by CsLOX3 mainly to 15-hydroperoxy-5,8,11,13-eicosatetraenoic acid (15-HPETE).

## 3. Discussion

The initial observations of 16-HOT occurrence were made when studying the GC-MS profiles of oxylipins (Me/TMS) in cucumber leaves, flowers, and fruit peels. Surprisingly, 16-HOT (Me/TMS) was the most abundant in these tissues. In view of these findings, the elucidation of the biosynthetic origin of 16-H(P)OT seemed to be an intriguing research goal. In vitro incubations of α-linolenic acid with cell-free preparations from flowers, leaves, and fruit peels resulted predominantly in the formation of 16(*S*)-HPOT. The identification of 16(*S*)-HPOT was confidently confirmed by the GC-MS analyses of Me/TMS derivatives of (1) the NaBH_4_-reduced products and (2) the NaBH_4_-reduced and totally hydrogenated products, as well as by (3) the NMR spectra of both 16(*S*)-HPOT (Me) and 16(*S*)-HOT (Me). The specific oxygenation of α-linolenate to 16(*S*)-HPOT signified the presence of some lipoxygenase isoenzyme controlling this conversion. To identify the protein, proteomic analyses were performed. The targeted proteomic analyses of cucumber flowers, leaves, and fruit peels revealed the presence of three LOX2 (Gene ID: 105434554) isoforms sharing more than 90% AA sequence identity. All of them are deposited at NCBI under the name “linoleate 9S-lipoxygenase 6-like”. One related gene (NP_001292637.1) expressed in the roots was cloned before [22]. This recombinant protein utilized α-linolenic acid as a preferred substrate, but its conversion products were not studied. Linoleic acid was reported to be non-specifically oxidized to a mixture of (*Z*,*E*) and (*E*,*E*) isomers of 13- and 9-HPODs [22].

The sequence named CsLOX3 was detected in the present work in cucumber leaves and fruit peels. CsLOX3 possessed 99.77% identity with the previously described isoenzyme NP_001292637.1. The recombinant CsLOX3 obtained by molecular cloning was most active toward α-linolenic acid and specifically converted it to 16(*S*)-HPOT. Additional tested ω3 fatty acids—eicosapentaenoic and hexadecatrienoic ones—were also oxidized by CsLOX3 to the corresponding ω3(*S*)-hydroperoxides, 18(*S*)-HPEPE and 14(*S*)-HPHT, respectively. To our knowledge, no lipoxygenases specifically dioxygenating the ω3 fatty acids at the ω3 position have been described before.

**Figure 7:**
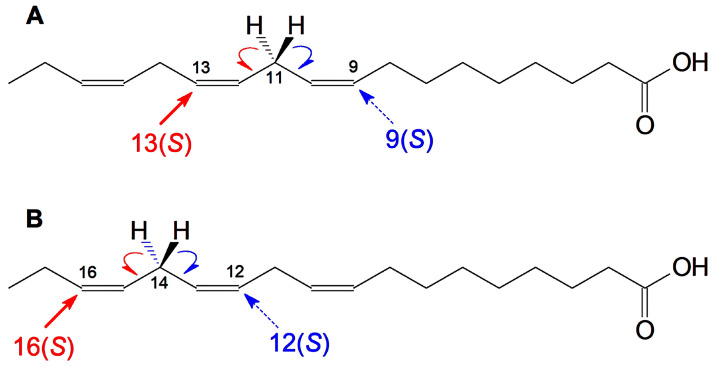
Specificity of α-linolenic acid oxygenation by conventional LOXs (**A**) and LOXs attacking the prochiral center C14 (**B**). Two prochiral centers (C11 and C14) of α-linolenic acid and the LOX specificity depending on selectivity of the initial hydrogen abstraction from C11 or C14.

The LOX substrate requirement is the presence of at least two methylene-interrupted double bonds [1,2,3]. The initial stage of LOX catalysis is the stereospecific hydrogen atom abstraction from the prochiral center (a methylene group between two double bonds). Although α-linolenic acid has two prochiral centers, C11 and C14 (Figure 7), only the C11 one was supposed to be attacked by LOXs. LOXs were considered to oxygenate both linoleic and α-linolenic acids at positions C13 or C9 only. Other hydroperoxides, like 12-HPOT or 16-HPOT (formed via the initial hydrogen abstraction from prochiral center C14), were considered the products of non-enzymatic fatty acid peroxidation. Probably, the only known exceptions yet are the LOXs of the blue-green algae *Acaryochloris marina* [16] and *Cyanothece* sp. [17], which oxidize the α-linolenic acid to 12(*R*)-HPOT, as well as the LOX of the δ-proteobacterium *Myxococcus xanthus*, which converts α-linolenate to 12(*S*)-HPOT [18]. As mentioned in the Introduction, the enantiopure 12(*S*)-(12–14), 12(*R*)-(9, 10), 16(*S*)-(15), and 16(*R*)-HOTs [12,13] have been detected in some plant species. A blast-disease-resistant rice cultivar possessed 16(*S*)-HOT [15]. However, no LOX capable of oxidizing α-linolenate to 16-HPOT formation has been identified yet.

CsLOX3 is the first LOX to oxidize α-linolenic acid to 16-hydroperoxide. Furthermore, CsLOX3 presents the first example of LOX catalyzing the ω3 oxygenation of ω3 fatty acids, namely α-linolenic acid. The only example of ω3 oxygenation described before is the eicosapentaenoic acid oxidation to 18(*R*)-hydroperoxide, a precursor of anti-inflammatory E-series resolvins in humans [4,5]. However, in the case of resolvin biosynthesis, ω3 oxygenation is controlled by either aspirin-pretreated cyclooxygenase-2 or microbial P450, but not lipoxygenase [4].

The ω3-hydroxylated oxylipins have been shown to possess physiological importance. So, aspirin-acetylated cyclooxygenase-2 (COX2) oxidizes eicosapentaenoic acid to 18-HEPE, the precursor of series E resolvins [23], the mediators of inflammation resolution. Related derivatives of α-linolenic acid, like 16-HOT, possess toxicity against fungal plant pathogens, such as the rice blast fungus *Magnaporthe grisea* [24]. 16-HOT also inhibits the growth of Gram-positive bacteria [25]. 16-HOT also displays anti-inflammatory activity toward TPA-induced edema in mouse ears [26].

**Figure 8:**
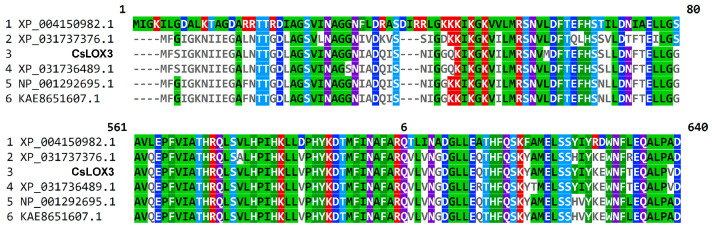
Selected multiple alignments of CsLOX3 and related cucumber proteins. All genes encoding proteins shown are localized in chromosome 2 of *C. sativus*. Most of them (except XP_004150982.1) share 90–98% identity.

**Figure 9:**
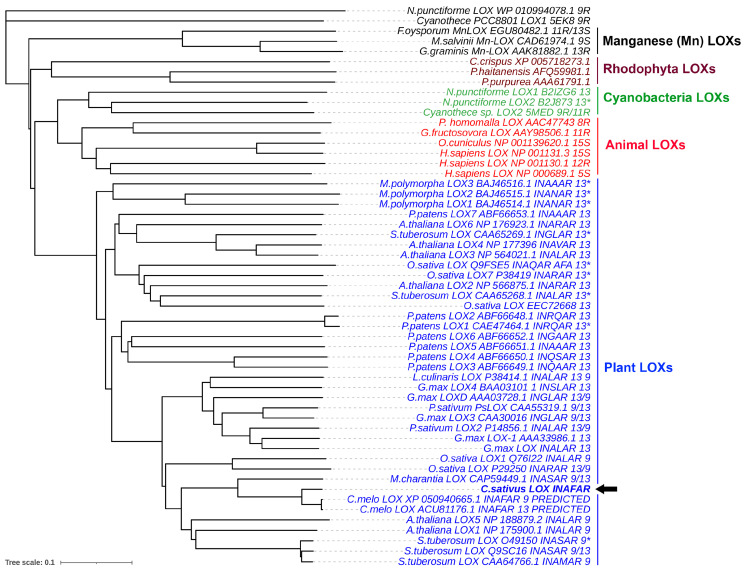
Phylogenetic tree of amino acid sequences of the selected studied LOXs from red algae, cyanobacteria, fungi, plants, and animals. Numbers 9 and 13 indicate the substrate specificity of LOXs determined using linoleic or linolenic acid. The numbers with an asterisk indicate enzymes characterized using both linoleic and linolenic acids. The black arrow indicates the CsLOX3 studied in the present work.

Higher plant genomes possess numerous genes encoding the putative “9S-LOXs types 6 and 5” related to *CsLOX3*. Cucumber, for instance, possesses several nearly identical sequences (Figure 8). All these isoenzymes contain the INAFAR motif within the catalytically essential α-helix 11, which has been proposed to be a part of the oxygen access channel [27]. Most of these proteins await biochemical characterization. If ever a few LOXs of this kind were cloned until now [22,28,29], their specificity was examined only with linoleic acid as a substrate (Figure 9). It seems that further studies in this direction might uncover the unidentified directions of the lipoxygenase pathway and oxylipin biosynthesis.

Our search using the NCBI and Phytozome databases uncovered 30 sequences containing PLAT and lipoxygenase domains at the N- and C-termini, respectively, in the cucumber genome. These two domains are characteristic of LOXs. Analysis of cucumber LOX genes revealed less transcript length variation (range 2199 to 2786 bp) compared to gene length variation (range 3580 to 14,270 bp). Exon–intron structure analyses depicted the presence of a varied number of introns at the cucumber LOX genes (6–8). The sizes of exons, introns, and UTRs in Supplementary Figure S2 are plotted in proportion to the length of their sequences. Most of the genes are localized on chromosomes 2 and 4 in the form of clusters, while the only genes are localized on chromosomes 5 and 6, and two genes are localized on chromosome 7. The presence of 30 LOX genes in the cucumber genome suggests that CsLOX3 may not be the only 16(*S*)-LOX in cucumber. Thus, 16(*S*)-LOX of cucumber flowers is still to be discovered.

The discovery of 16(*S*)-LOX as well as ω3(*S*)-LOX adds a new dimension to the knowledge of LOX catalytic capabilities. The 16(*S*)-HPOT may play the role as a substrate of different CYP74 enzymes and, thus, a precursor of some currently unknown oxylipins. To conclude, the observations in the present work raise new questions. Firstly, it has to be clarified how widespread 16- and 12-LOXs are in plants and other phyla. Secondly, the metabolism of 16- and 12-HPOTs, as well as the physiological importance thereof and their metabolites, has to be studied. These seem to be the important goals of future work.

## 4. Materials and Methods

### 4.1. Plant Material

Cucumber seeds (variety “Malyshok”, purchased from agricultural holding Poisk, Moscow, Russia) after four days of germination were planted in the open soil on 8 April 2016. Leaves, flowers, and fruits were harvested on 9 August 2016, frozen under liquid nitrogen, and then stored at −80 °C before use. Fruits were also bought in the shops, and the cut peel was used for experiments.

### 4.2. Chemicals

Linoleic, α-linolenic, eicosapentaenoic, and 13,16-docosadienoic acids, as well as isopropyl β-D-thiogalactoside, silylating reagents, and NaBH_4_, were purchased from Sigma (St. Louis, MO, USA). (7*Z*,10*Z*,13*Z*)-hexadecatrienoic acid was generously provided by Larodan (Stockholm, Sweden). [^2^H_6_]Benzene (99.5% ^2^H) was acquired from the “Applied Chemistry Centre” (St. Petersburg, Russia). Oligonucleotides were obtained from Evrogen (Moscow, Russia).

### 4.3. In Vitro Incubations of α-Linolenic Acid with Crude Cucumber Enzyme Preparations

Cucumber peel (10 g) was homogenized with Ultra-Turrax, and the resultant fine powder was extracted three times at −10 °C with 250 mL of acetone. The pellet was dried in vacuo and stored at −20 °C. The enzyme solution was prepared by further extraction in Tris/HCl buffer (50 mM, 50 mL), pH 7.5, at 0 °C for 1 h. After precipitation of the pellet by centrifugation at 8000× *g* for 5 min at 4 °C, the resultant supernatant was decanted and used as an enzyme preparation for incubation. Fresh enzyme solutions were incubated (total volume 20 mL) with α-linolenic acid (1 mg) at 23 °C for 30 min under continuous oxygen bubbling.

Alternatively, cucumber flowers (2 g) or leaves (10 g) were suspended in cold Tris/HCl buffer (50 mM, 10 mL (flowers) or 20 mL (leaves)), pH 7.5, and homogenized with Ultra-Turrax. The homogenates were filtered through cheesecloth and centrifuged at 8000× *g* for 5 min at 4 °C. The supernatants were decanted and used for incubations with α-linolenic acid (200 μg (flowers) or 1 mg (leaves)) at 23 °C for 30 min under continuous oxygen bubbling.

The reaction mixture was acidified to pH 6.0, and the products were extracted with a hexane/ethyl acetate (1:1, by volume) mixture. After the in vacuo evaporation of solvents, the residue was dissolved in a chloroform/isopropanol (2:1, by volume) mixture. Acidic lipids were separated and purified using Supelclean LC-NH_2_ (3 mL) cartridges (Supelco, Bellefonte, PA, USA). Firstly, the total lipid extract dissolved in a solvent mixture of chloroform and isopropanol (2:1, by volume) was passed through the cartridges. Free carboxylic acids were eluted with the solvent mixture ethyl acetate/acetic acid (98:2, by volume). Then, the products were methylated with ethereal diazomethane and trimethylsilylated with a pyridine/hexamethyldisilazane/trimethylchlorosilane (2:1:2, by volume) mixture at 23 °C for 15 min. The silylation reagents were evaporated in vacuo. The dry residue was dissolved in 100 μL of hexane and analyzed using GC-MS. When specified, the products were reduced with NaBH_4_, methylated, hydrogenated over PtO_2_, trimethylsilylated, and analyzed using GC-MS.

For the extraction of oxylipins from cucumber leaves, flowers, and fruit peels, the tissues (5 g fresh weight) were homogenized at 0–4 °C and extracted with a cold (0–4 °C) hexane/ethyl acetate (1:1, by volume) mixture. The following procedures were the same as those described in the previous paragraph.

### 4.4. Product Analyses

The products were purified first using RP-HPLC on a Macherey–Nagel Nucleosil 5 ODS column (250 × 4 mm, 5 µm) using the solvent mixture methanol/water (linear gradient from 76:24 to 96:4, by volume) at a flow rate of 0.4 mL/min. The peaks of the products were collected and purified using NP-HPLC on a Macherey–Nagel Nucleodur 100–3 column (250 × 4.6 mm, 3 μm) using the solvent mixture hexane/isopropanol (98.5:1.5, by volume) at a flow rate of 0.4 mL/min. After NaBH_4_ reduction, the final purification of the products was performed using NP-HPLC as described above. Product enantiomers (methyl esters) were separated on a Chiralcel OD-H column (250 × 4.6 mm, 5 µm) with hexane/isopropanol (97:3, by volume) at a flow rate of 0.4 mL/min.

### 4.5. Isolation and Identification of Products

The products (Me esters) were separated using RP-HPLC on a Macherey–Nagel Nucleosil 5 ODS column (250 × 4 mm, 5 μm) using the solvent mixture methanol/water (linear gradient from 60:40 to 96:4, by volume) at a flow rate of 0.4 mL/min. The peaks of the products were collected and purified using NP-HPLC, as described in the previous section. All HPLC analyses were performed with a Shimadzu LC-20AB solvent delivery pump with UV spectral monitoring (190–370 nm) using a Shimadzu SPD-M20A diode array detector (Shimadzu, Kyoto, Japan). Separate products were collected after NP-HPLC separation, redissolved in [^2^H_6_]benzene, and then, the NMR spectra were recorded. For additional qualitative information, the products (Me/TMS) were re-analyzed using GC-MS after hydrogenation over PtO_2_ or after sequential NaBH_4_ reduction, hydrogenation, methylation, and trimethylsilylation.

Product **1** collected after the NP-HPLC purification was dissolved in [^2^H_6_]benzene and subjected to the NMR spectral records. Then, product **1** was reduced with NaBH_4_ to compound **2**, which was finally purified using NP-HPLC, as described in the previous section. Then, product **2** was dissolved in [^2^H_6_]benzene, and its NMR spectra were recorded.

### 4.6. The Targeted Proteomic Analyses of Cucumber Flowers and Fruit Peel Samples

Proteins of cucumber flowers, leaves, and fruit peels (1 g) were extracted with 25 mM Tris-HCL buffer, pH 8.0, containing 100 mM DTT, 50 mM EDTA, and protease inhibitors (1%) for 1 h at 4 °C; then centrifuged for 20 min at 20,000× *g*; separated using 1D SDS-PAGE (12.5% acrylamide); and stained with Coomassie Brilliant Blue G-250. The protein bands corresponding to the expected LOX molecular masses (95–100 kDa) were excised and subjected to tryptic digestions in accordance with a previous method [30]. LC–ESI–MS/MS analyses of products of trypsinolysis were performed using a C18 Acclaim PepMap 100 (150 mm, 75 µm I.D., 3 µm particle size) analytical column (Thermo Scientific, Waltham, MA, USA) on a nano-HPLC UltiMate 3000 (Dionex (Thermo Scientific), The Netherlands) online coupled to a MicrOTOF-Q (Bruker Daltonics, Bremen, Germany) mass spectrometer, as described before [31]. Mascot Deamon software version 2.2.6 (Matrix Science Inc., London, UK) was used for the MS/MS spectra search. The following searching parameters were applied: precursor ion mass tolerance of 0.1 Da, fragment ion mass tolerance of 0.1 Da, one missed cleavage, and possible variable modifications (carbamidomethylation of cysteines and oxidation of methionine). Hits were considered reliable if at least two peptides were matched in Mascot analysis with an ion score that indicated identity or extensive homology (*p* < 0.05). The identification of target proteins was reliable, with a total Mascot score of 1150–1890 and a protein sequence coverage of 23–50%.

### 4.7. Bioinformatics and Phylogenetic Analysis

*Cucumis sativus* genomic data at the NCBI database (https://www.ncbi.nlm.nih.gov/genome/annotation_euk/Cucumis_sativus/102/, accessed on 1 February 2023, assembly name Cucumber_9930_V3), as well as the Phytozome database (https://phytozome-next.jgi.doe.gov/info/Csativus_v1.0, accessed on 1 February 2023), were used to retrieve the gene and protein sequences for the putative LOXs. Multiple alignments of the amino acid and nucleotide sequences were performed using MEGA7 software (version 7.0.26) [32]. The molecular phylogenetic relationships and gene structures of cucumber LOXs were shown using the Gene Structure Display Server (GSDS) online tool (http://gsds.gao-lab.org/, accessed on 1 June 2023) [33]. Chromosomal locations of cucumber LOX genes were obtained from the Genome Data Viewer resource of the NCBI database (https://www.ncbi.nlm.nih.gov/genome/gdv/?org=cucumis-sativus, accessed on 1 June 2023, assembly name Cucumber_9930_V3). Primer construction was performed using Vector NTI Advance 11.5 software (Invitrogen, San Diego, CA, USA). The search for chloroplast-targeting sequences was performed using TargetP–2.0 Prediction Server (https://services.healthtech.dtu.dk/services/TargetP-2.0/, accessed on 1 June 2023). The evolutionary history was inferred using the minimum-evolution method [34]. The optimal tree with a sum of branch lengths of 12.58771715 is shown. The bootstrap consensus tree was inferred from 1000 replicates [35]. The tree is drawn to scale, with branch lengths in the same units as those of the evolutionary distances used to infer the phylogenetic tree. The evolutionary distances were computed using the p-distance method [36] and were in units of the number of amino acid differences per site. The ME tree was searched using the close-neighbor-interchange algorithm [36] at a search level of 1. The neighbor-joining algorithm [37] was used to generate the initial tree. The analysis involved 55 amino acid sequences. All ambiguous positions were removed for each sequence pair. There were a total of 1328 positions in the final dataset. Evolutionary analyses were conducted in MEGA7 [32]. The iTOL tool (https://itol.embl.de/, accessed on 1 June 2023) was used to visualize the phylogenetic model output.

### 4.8. Molecular Cloning of Cucumber CsLOX3 Gene

The total RNA was isolated from the leaves of *C. sativus* plants. The primers for cloning the open reading frame (ORF) of the *CsLOX3* gene were constructed using the NCBI GeneID: 101,206,086 gene sequence. The cDNA obtained after the reverse transcription was used as the template for PCR with the constructed primers CsLOX_F GACGACGACAAGATGTTTTCAATTGGGAAGAATATC and CsLOX_R GAGGAGAAGCCCGGAATAGAGATGCTGTTAGGAATTCC. The ca. 2700-bp product obtained using PCR was initially cloned into the pGEM-T vector (Promega, Madison, WI, USA) and sequenced to verify the presence of the CsLOX3 fragment. Then, the ORF of the *CsLOX* gene was subcloned into the expression pET-32 Ek/LIC vector using a method of ligation-independent cloning.

### 4.9. Expression and Purification of Recombinant CsLOX3 Enzyme

The resulting construct was transferred into *Escherichia coli* Rosetta-gami(DE3)pLysS B host cells. The recombinant gene was expressed in host cells as follows: an overnight culture (10 mL) of bacteria was inoculated into 1 L of Luria–Bertani medium supplemented with one volume of mineral medium M9. Bacteria were grown at 37 °C and 250 rpm to an OD_600_ of 0.6. Expression of the target gene was induced by the addition of 0.5 mM isopropyl-β-D-1-thiogalactopyranoside to the medium. Induction was controlled using 12.5% SDS-PAGE analysis of crude cellular extracts. Bacteria were harvested using centrifugation for 15 min at 4500 rpm at 4 °C and lysed with BugBuster Protein Extraction Reagent (Novagen, Madison, WI, USA). For the purification of the recombinant CsLOX3 from the cell lysate, anion-exchange chromatography was performed on a BabyBio Q 1 mL cartridge (Bio-Works, Uppsala, Sweden) and an NGC Discover 10 chromatographic system (Bio-Rad, Hercules, CA, USA). The protein was eluted with a linear gradient of NaCl (10–700 mM) in 50 mM Tris-HCl buffer (pH 7.5). The elution was monitored by absorption at 280 nm. The presence of CsLOX3 in the collected fractions was determined using 12.5% SDS-PAGE electrophoresis. Protein bands in gel were stained with Coomassie Brilliant Blue G-250. To confirm the presence of CsLOX3, the band of interest was cut from the gel and analyzed using HPLC-MS with a MicrOTOF-Q mass-spectrometer (Bruker Daltonics, Bremen, Germany), as described above.

### 4.10. Kinetic Studies of Recombinant CsLOX3

The enzymatic activity of the purified recombinant CsLOX3 was determined by monitoring the 234 nm signal increase with a PB 2201 B UV-VIS spectrophotometer (SOLAR, Minsk, Belarus) with substrate concentrations ranging from 5 to 150 μM. The measurements were carried out in 0.6 mL of Na-phosphate buffer (pH 7.5) at 25 °C. Kinetic parameters were calculated by fitting the datasets to a one-site saturation model for simple ligand binding using SigmaPlot 11 software (Systat Software Inc., Palo Alto, CA, USA). Five independent experiments were performed for each specified variant.

### 4.11. Micropreparative Isolation and Purification of Selected Products

The standard assay mixture (2 mL), containing 0.0032% Tween 20 and 300 μM linoleate, α-linolenate, arachidonate, hexadecatrieonate, or eicosapentaenoate in 50 mM sodium phosphate buffer (pH 7.5), was preliminary bubbled with oxygen for 3 min. The reaction was started by the addition of purified CsLOX3 preparation (0.028 U) and proceeded for 40 min at 23 °C. Enzymatic activity was determined by monitoring the increase in absorbance at 234 nm with a Spekol 1200 (Analytic Jena, Jena, Germany) UV/VIS spectrophotometer. The products (Me esters) were separated using RP- and NP-HPLC, as described above. For additional qualitative information, the products (Me or Me/TMSi) were re-analyzed using GC-MS after the hydrogenation over PtO_2_ or after the sequential NaBH_4_ reduction, hydrogenation, methylation, and trimethylsilylation, as described above.

### 4.12. GC-MS Analyses

Products (Me esters or Me/TMS derivatives) were analyzed using GC-MS with a Shimadzu QP2020A mass spectrometer connected to a Shimadzu GC-2010 Plus gas chromatograph equipped with a Macherey–Nagel Optima-5-MS (5% phenyl, 95% methylpolysiloxane) fused capillary column (length, 30 m; ID, 0.25 mm; film thickness, 0.25 μm). Helium at a linear velocity of 30 cm/s was used as the carrier gas. Injections were made in split mode using an initial column temperature of 120 °C and an injector temperature of 230 °C. Then, the column temperature was raised by 10 °C/min until 240 °C. Electron impact ionization (70 eV) was used.

### 4.13. NMR Spectroscopy

The ^1^H-NMR (600 MHz), ^1^H-^1^H-COSY (600/600 MHz), ^1^H-^1^H-TOCSY (600/600 MHz), ^1^H-^13^C-HSQC (600/151 MHz), and ^1^H-^13^C-HMBC (600/151 MHz) spectra ([^2^H_6_]benzene, 303 K) were recorded with a Bruker Avance III 600 spectrometer.

## Figures and Tables

**Table 1:** The NMR spectral data for compound **2** (Me ester), [^2^H_6_]benzene, 303 K. The ^1^H-NMR (600 MHz), ^1^H-^1^H-COSY (600/600 MHz), ^1^H-^1^H-TOCSY (600/600 MHz), ^1^H-^13^C-HSQC (600/151 MHz), and ^1^H-^13^C-HMBC (600/151 MHz) data are presented.

Position Number	^13^C Chemical Shifts (ppm), Functional Group	^1^H Chemical Shifts (ppm), Multiplicity, Coupling Constant (Hz)	2D NMR Correlations		
			COSY	TOCSY	HMBC
1	173.74, COOMe				COOMe
2	34.04, CH_2_	2.10, t, 7.5 (H3)	H3	H3, H4	C1, C3, C4
3	25.17, CH_2_	1.54, m	H2, H4	H2, H4–H8	C1, C2, C4
4	29.0–29.5, CH_2_	1.16, m	H3	H2, H3, H7, H8	C2, C3
5	29.0–29.5, CH_2_	1.15–1.20, m			
6	29.20, CH_2_	1.16, m	H7	H2–H11	
7	29.68, CH_2_	1.27, m	H6, H8	H2–H6, H8–H11	C8, C9
8	27.43, CH_2_	2.01, m	H7, H9	H3–H7, H9–H11	C7, C9, C10
9	131.15, CH	5.42–5.46, AB	H8, H10	H6–H8, H11–H131	C7, C8, C11
10	127.58, CH	5.42–5.46, AB	H8, H9, H11	H6–H9, H11–H14	C8, C11, C12
11a	26.46, CH	2.87–2.98, AB	H9, H10, H12, H15	H6–H10, H12, H13–H16	C9, C10, C12, C13
11b		2.87–2.98, AB			
12	131.15, CH	5.44, m	H11, H13	H11, H13–H15	C10, C11, C14
13	128.41, CH	6.02, dddt, 11.1 (H14), 10.9 (H12), 0.8 (H15), 1.6 (H11a,b)	H12, H14	H11, H12, H14–H16	C11, C14, C15
14	129.11, CH	6.65, dddd, 15.3 (H15), 11.1 (H13), 1.0 (H12), 1.0 (H16)	H13, H15	H11–H13, H15–H18	C12, C13, C15, C16
15	133.08, CH	5.54, dd, 15.3 (H14), 7.8 (H16)	H11, H14, H16	H12–H14, H16–H18	C13, C16, C17
16	87.43, CH	4.24, dddd, 7.8 (H15), 7.0 (H17a), 6.8 (H17b), 0.9 (H14)	H15, H17, H18	H14, H15, H17, H18	C14, C15, C17, C18
17a	25.98, CH_2_	1.43, ddq, 14.1 (H17b), 7.0 (H16), 7.5 (H18)	H16, H18	H13–H18	C15, C16, C18
17b		1.63, ddq, 14.1 (H17a), 6.8 (H16), 7.5 (H18)			
18	9.68, CH_3_	0.84, t, 7.5 (H17a,b)	H16, H17	H16, H17a, 17b	C16, C17
(1)	50.98; COOMe	3.36, s			COOMe

**Table 2:** The NMR spectral data for compound **1** (Me ester), [^2^H_6_]benzene, 303 K. The ^1^H-NMR (600 MHz), ^1^H-^1^H-COSY (600/600 MHz), ^1^H-^1^H-TOCSY (600/600 MHz), ^1^H-^13^C-HSQC (600/151 MHz), and ^1^H-^13^C-HMBC (600/151 MHz) data are presented.

Position Number	^13^C Chemical Shifts (ppm), Functional Group	^1^H Chemical Shifts (ppm), Multiplicity, Coupling Constant (Hz)	2D NMR Correlations		
			COSY	TOCSY	HMBC
1	173.62, COOMe				COOMe
2	34.04, CH_2_	2.11, t, 7.5 (H3)	H3	H3, H4	C1, C3, C4
3	25.17, CH_2_	1.55, m	H2, H4	H2, H4–H8	C1, C2, C4
4	29.0–29.6, CH_2_	1.16, m	H3	H2, H3, H7, H8	C2, C3
5	29.0–29.6, CH_2_	1.15–1.20, m			
6	29.36, CH_2_	1.19, m	H7	H2, H3, H4–11	
7	29.68, CH_2_	1.28, m	H6, H8	H2–H6, H8–H11	C8, C9
8	27.42, CH_2_	2.02, dt, 7.0 (H7), 7.0 (H9)	H7, H9	H3–H7, H9–H11	C7, C9, C10
9	130.99, CH	5.43–5.46, AB	H8, H10	H6–H8, H11–H13	C8, C11
10	127.76, CH	5.43–5.46, AB	H8, H9, H11	H6–H9, H11–H14	C8, C11
11	26.46, CH_2_	2.97, m	H9, H10, H12, H15	H6–H10, H12–H16	C9, C10, C12, C13, C14
12	130.18, CH	5.45, m	H11, H13	H11, H13–H15	C11, C14
13	128.73, CH	6.04, dddt, 11.0 (H14), 10.9 (H12), 0.8 (H15), 1.6 (H11a,b)	H12, H14	H11, H12, H14–H16	C14, C15
14	125.34, CH	6.60, dddd, 15.2 (H15), 11.0 (H13), 1.2 (H12), 1.2 (H16)	H13, H15	H11–H13, H15–H18, CHOH	C12, C13, C16
15	137.44, CH	5.58, dd, 15.2 (H14), 6.3 (H16)	H11, H14, H16	H12–H14, H16–H18, CHOH	C12, C13, C16, C17
16	73.72, CH	3.88, m	H15, H17, H18	H11, H14, H15, H17, H18, CHOH	
17a	30.65, CH_2_	1.41, m	H16, H18	H14–H18, CHOH	C15, C16, C18
17b		1.46, m			
18	9.68, CH_3_	0.85 t, 7.5 (H17a,b)	H16, H17	H15, H16, H17, CHOH	C16, C17
(1)	50.81; COOMe	3.35, s			COOMe
(16)	CHOH	1.04, d, 4.1 (H16)		H14–H18	

**Table 3:** Kinetic parameters and substrate specificity of recombinant CsLOX3.

Substrate	*k_cat_* (s^−1^)	*K_M_* (μM)	*k_cat_*/*K_M_* (μM^−1^∙s^−1^)	Specificity (%)
18:2, linoleic acid	46	21	2.2	30.6
18:3, α-linolenic acid	43	6	7.2	100

## Data Availability

All described data are contained within the article and Supplementary Materials.

## Supplementary Materials

The following supporting information can be downloaded at: https://www.mdpi.com/article/10.3390/ijms241612977/s1.
