# Supplementary material for: Discovery of α-Linolenic Acid 16(S)-Lipoxygenase: Cucumber (Cucumis sativus L.) Vegetative Lipoxygenase 3
Source: Int J Mol Sci. 2023 Aug 19;24(16):12977. doi: 10.3390/ijms241612977 (PMC10454662; doi:10.3390/ijms241612977)
Supplement: Supplementary file 1 [file ijms-24-12977-s001.zip › ijms-2541424-supplementary.pdf]

**Supplementary Information**

Supplementary Table S1. The list of identified LOXs in *Cucumis sativum*.

| Analyzed *Cucumis sativum* organ (or tissue) | Protein ID in NCBI | Protein sequence, matched peptides shown in **bold red.** | Mascot Score | Sequence coverage, % |
|---|---|---|---|---|
| Peel | KGN60957.2 | **1** MFSIGKNIIE GALNTTGDLA GSVINAGGNI ADQISNIGGQ KIKGKVILMR<br>**51** SNVLDFTEFH SSLLDNFTEL LGGGVSLQLI SATQTSALDS RGKVGKKAFL<br>**101** ERWLTSIPPL FAGESVFQVS FTWEEGFGFP GAFFIKNGHT SEFFLKSLTL<br>**151** EDVPGFGHVH FDCNSWVYPS GRYKKDR**IFF** **ANNTYLPSDT** **PNPLRKYREE**<br>**201** **ELLTLR**GDGT GERKEWDR**IY** **DYDIYNDLSE** **PGDGRPILGG** **SQFPYPR**RGR<br>**251** TGRPREWKDS NYESRLPVVS GLNIYVPRDE NFGHLK**LSDF** **LGFALK**SLVA<br>**301** TVQPALVNIV DFTPGEFDKF QDVHNLYEGG LPVPLDVFRN LTK**DFTPPMF**<br>**351** **QELLR**TDNDQ RFLKFSPPQV VKEDK**FAWQT** **DEEFAREMLA** **GVNPLIIR**RL<br>**401** KEFPPKSKLD PKMYGDQHSK ITEEDIK**SGL** **EGLTVAEALN** **QK**R**LYILDHH**<br>**451** **DALMPYLR**KI NSTKTKTYAT RTLLLLKNDG TLKPLVIELS LPHPQGDQFG<br>**501** ANSKQYFPAE EGVQKSIWQL AKAYVVVNDA GYHQLISHWL NTHAVQEPFV<br>**551** IATHRQLSVL HPIHKLLVPH YK**DTMFINAF** **ARQVLVNGDG** **LLEQTHFQSK**<br>**601** YAMELSSYIY KEWNFTEQAL PVDLIKRGVA VEDPSSPNGV K**LLIEDYPFA**<br>**651** **VDGLEIWSTI** **K**TWVTNYCSL YYKDDSAIQN DVELQSWWKE VREKGHVDKK<br>**701** NETWWPKLQN FNELVETCTT IIWISSALHA AVNFGQYPYG GFMPNRPTIS<br>**751** RRLIPEVGSA EYKELESKPE KAYLKTVNSM LQTLLGVSLI EILSRHASDE<br>**801** VYLGQRASIE WTSDKAAVEV FENFGKKVFE VESRIIERNK DVNLKNR**SGP**<br>**851** **VNVPYTLLLP** **SSTEGLTGR**G IPNSISI | 1018 | 23 |
| | XP_011648829.1 | **1** MFGIGKNIIE GALNTTGDLA GSVLNAGGNI VDKVSSIGDK KIKGKVILMR<br>**51** SNVLDFTQLH SSVLDTFTEI LGSGVTFQLI SATQASFDSR GKVGKKAFLE<br>**101** KWITSIPPLF AGESLFEVNF SWDDSNFGYP GAFYVQNGHT SHFFLKSLTL<br>**151** EDVPGYGRVH FDCNSWVYPS GRYKKDRIFF ANNTYLPKDT PNPLRKYREE<br>**201** ELLNLRGDGT GERKEWDR**IY** **DYDLYNDISE** **PGDGRPILGG** **SK**YPYPRRGR<br>**251** TGRQRHWKDS NYESR**LSVVS** **GLNIYVPR**DE NFGHLK**LSDF** **LGFALK**SLAS<br>**301** TVFPALINIV NITRPGGEFE KFQDVHDLYE GGLPVPLDVF RNLTK**DFTPP**<br>**351** **MFQELLR**TDN DQRFLKFSPP QVVKGILYIY XXDEEFAR**EM** **LAGVNPLIIR** | 716 | 22 |

| | | | | |
|---|---|---|---|---|
| | | 401 RLEVFPPKSK LDPNIYGDQH SKITEKDIK**S** **GLEGLTVDEA** **LNQR**KLFTLD<br>451 HHDALMPYLR KINSTKTKAY ATRTLLFLK**D** **DGTLKPLVIE** **LSLPHPQGDQ**<br>501 **FGANSK**QYFP AEEGVQKSIW QLAKAYVVVN DAGYHQLISH WLHTHAVQEP<br>551 FVIATHRQLS ALHPIHKLLV PHYK**DTMFIN** **AFARQVLVNG** **DGLLEQTHFQ**<br>601 **SK**YAMELSSH IYKEWNFREQ ALPADLIKRG VAVEDARSTY GLK**LLIEDYP**<br>651 **FAVDGLEIWS** **TIK**TWVTNYC SLYYKDDSAI QNDVELQSWW KEVREKGHAD<br>701 KKNESWWPKL QNFDELVETC TTIIWVSSAL HAAVNFGQYP YGGFMPNRPT<br>751 ISRRHMPEVG SAEYKELESK PEKAYLKTVN SMLQTLLGVS LIEILSRHAS<br>801 DELYLGERAS TEWTSEKDAL ELCEYFGK**AM** **SEVESNIIER** NKDVNLKNR**T**<br>851 **GPVNVPYTLL** **LPSSAEGLTG** **R**GIPNSISI | | |
| | NP_001292695.1 | 1 MFGIGK**NIIE** **GAFNTTGDLA** **GSVINAGGNI** **ADQISNIGGK** KIKGKVILMR<br>51 SNVLDFTEFH SNLLDNFTEL LGGGVSIQLI SATQTSALES RGKVGKKAFL<br>101 ERWLTSIPPL FAGESVFQVS ITWEDGFGFP GAFFIRNGHT SEFFLK**SLTL**<br>151 **EDVPGVGSVH** **FDCNSWVYPS** **GR**YKKDR**IFF** **ANNTYLPSDT** **PNPLR**KYREE<br>201 ELLTLRGDGT GERKEWDR**IY** **DYDIYNDISE** **PGDGRPILGG** **SQFPYPR**RGR<br>251 TGRPRERKDS NYESR**LSVVS** **GLNIYVPR**DE NFGHLK**LSDF** **LGFALKSLVS**<br>301 **TVQPALLNIV** **NITRPGGEFD** **KFQDVHNLYE** **GGLPVPLNVF** **R**NLTK**DFTPP**<br>351 **MFQELLR**TES DQRFLKFSPP QVVKHDK**SAW** **LTDEEFAREM** **LAGVNPLIIR**<br>401 GLEEFPPKSK LDPKLYGDQH SKISEEDIKF GLEGLTVAEA LNQKK**LYILD**<br>451 **HHDALMPYLR** KINSTKTKAY ATRTLLLLK**D** **DGTLKPLVIE** **LSLPHPQGDQ**<br>501 **FGANSK**QYFP AEEGVQKSIW QLAKAYVVVN DAGYHQLISH WLNTHAVQEP<br>551 FVIATHRQLS VLHPIHKLLV PHYK**DTMFIN** **AFARQVLVNG** **DGLLEQTHFQ**<br>601 **SK**YAMELSSH VYK**EWNFLEQ** **ALPADLIK**RG VAVEDASSPH GLK**LLIEDYP**<br>651 **FAVDGLEIWS** **TIK**TWVTNYC SLYYKDDNAI R**NDVELQSWW** **K**EAREKGHAD<br>701 KKNETWWPKL QNFNELVEAC TTIIWISSAL HAAVNFGQYP YGGFIPNRPT<br>751 ISRRHMPEVG SAEYKELESK PEKAYLKTVN SMLQTLLGVS LIEILSRHAS<br>801 DEVYLGQRDS IKWTSDKDAI ERFEKFGKDM YDVESRIIER NKDGNLKNR**S**<br>851 **GPVNVPYTLL** **LPSSTEGLTG** **R**GIPNSISI | 1396 | 39 |
| Flowers | NP_001292695.1 | 1 MFGIGK**NIIE** **GAFNTTGDLA** **GSVINAGGNI** **ADQISNIGGK** **K**IKGKVILMR<br>51 SNVLDFTEFH SNLLDNFTEL LGGGVSIQLI SATQTSALES RGKVGKKAFL<br>101 ERWLTSIPPL FAGESVFQVS ITWEDGFGFP GAFFIRNGHT SEFFLK**SLTL**<br>151 **EDVPGVGSVH** **FDCNSWVYPS** **GR**YKKDR**IFF** **ANNTYLPSDT** **PNPLRKYREE**<br>201 **ELLTLR**GDGT GERKEWDR**IY** **DYDIYNDISE** **PGDGRPILGG** **SQFPYPR**RGR | 1890 | 50 |

| | | | | |
|---|---|---|---|---|
| | | 251 TGRPRERKDS NYESR**LSVVS** **GLNIYVPR**DE NFGHLK**LSDF** **LGFALKSLVS**<br>301 **TVQPALLNIV** **NITRPGGEFD** **KFQDVHNLYE** **GGLPVPLNVF** **R**NLTK**DFTPP**<br>351 **MFQELLR**TES DQRFLKFSPP QVVKHDK**SAW** **LTDEEFAREM** **LAGVNPLIIR**<br>401 GLEEFPPKSK LDPKLYGDQH SK**ISEEDIKF** **GLEGLTVAEA** **LNQKKLYILD**<br>451 **HHDALMPYLR** KINSTKTKAY ATR**TLLLLKD** **DGTLKPLVIE** **LSLPHPQGDQ**<br>501 **FGANSKQYFP** **AEEGVQK**SIW QLAKAYVVVN DAGYHQLISH WLNTHAVQEP<br>551 FVIATHRQLS VLHPIHKLLV PHYK**DTMFIN** **AFARQVLVNG** **DGLLEQTHFQ**<br>601 **SKYAMELSSH** **VYKEWNFLEQ** **ALPADLIK**RG VAVEDASSPH GLK**LLIEDYP**<br>651 **FAVDGLEIWS** **TIK**TWVTNYC SLYYKDDNAI R**NDVELQSWW** **K**EAREKGHAD<br>701 KKNETWWPKL QNFNELVEAC TTIIWISSAL HAAVNFGQYP YGGFIPNRPT<br>751 ISRR**HMPEVG** **SAEYKELESK** **PEK**AYLKTVN SMLQTLLGVS LIEILSRHAS<br>801 DEVYLGQRDS IKWTSDKDAI ERFEK**FGKDM** **YDVESR**IIER NKDGNLKNR**S**<br>851 **GPVNVPYTLL** **LPSSTEGLTG** **R**GIPNSIS | | |
| | XP_004150982.1 | 1 MIGKILGDAL KTAGDARRTT R**DIAGSVINA** **GGNFLDR**ASD IRRLGKKKIK<br>51 GKVVLMRSNV LDFTEFHSTI LDNIAELLGS GIVINLVSAT EVDRDSNDPR<br>101 GKIGRRAFLE RWLTSLPPVF AGESVFQVNF EWEDDFGYPG AFYIKNGHTS<br>151 EFFLK**SLTLE** **DVPGYGR**VHF DCNSWVYPQR RYRKDRIFFA NKSWLPSETP<br>201 EPLRKYREEE LLNLRGDGKG ERQEWDR**IYD** **YDVYNDIADP** **DAGDKLVRPI**<br>251 **LGGSQYPYPR** RGRTGRPKTR RDPNSERRLQ SVIGLNIYVP RDENFGHLK**M**<br>301 **GDFLGYALK**A LSASVKPGLQ TVFDITPGEF DNYKEVHNLY EGGFPIPQTL<br>351 FKHLSDSLSA PLLKEVLRID GDRFLR**FAVP** **DVIKDDK**SAW RTDAEFAR**EM**<br>401 **IAGVNPILIS** **R**LEHFPPLSK LDPKR**YGNQN** **STITEEQIKD** **GLEGLSVQEA**<br>451 **MK**ENK**LYILD** **HHDALMPYLR** KINSTSTKTY ATRTLLFLK**D** **DGTLKPLVIE**<br>501 **LSLPHPQGDH** **LGAISK**LYFP VEKSNVEGSI WQLAKAYVAV NDAGYHQLIS<br>551 HWLNTHAVLE PFVIATHRQL SVLHPIHKLL DPHYKDTMFI NAFAR**QTLIN**<br>601 **ADGLLEATHF** **QSKFAMELSS** **YIYRDWNFLE** **QALPADLLK**R GVAIKDPSSP<br>651 HGLK**LLIEDY** **PYAVDGLEIW** **STIK**NWVADY CNIYYR**DDTA** **IQNDIELQSW**<br>701 **WK**EVVEKGHA DKKHEAWWPK MQTLNELIES CSIIIWIASA LHAAVNFGQY<br>751 AYGGFVPNRP TVSRTFMPEV GSKEYKELES CPEKAFLRTI NSQLQCLLGM<br>801 SLIEILSRHA SDEVYLGKRG SLEWTCDRDA LEAFDDFGQE VNEVEERIME<br>851 RNRNIKFKNR TGQANVPYTL LLPSSNEGIT GKGIPNSISI | 518 | 27 |
| Leaves | | 1 MFSIGKNIIE GALNTTGDLA GSVINAGGNI ADQISNIGGQ KIKGKVILMR | 195 | 7 |

| | | | | |
|---|---|---|---|---|
| | | **51** SNVMDFTEFH SSLLDNFTEL LGGGVSLQLI SATQTSALDS RGKVGKKAFL<br>**101** ERWLTSIPPL FAGESVFQVS FTWEEGFGFP GAFFIKNGHT SEFFLKSLTL<br>**151** EDVPGFGHVH FDCNSWVYPS GRYKKDRIFF ANNTYLPSDT PNPLRKYREE<br>**201** ELLTLRGDGT GERKEWDRIY DYDIYNDLSE PGDGRPILGG SQFPYPRRGR<br>**251** TGRPREWKDS NYESRLPVVS GLNIYVPRDE NFGHLK**LSDF LGFALK**SLVA<br>**301** TVQPALVNIV DFTPGEFDKF QDVHNLYEGG LPVPLDVFRN LTK**DFTPPMF**<br>**351** **QELLR**TDNDQ RFLKFSPPQV VKEDKFAWQT DEEFAR**EMLA GVNPLIIR**RL<br>**401** KEFPPKSKLD PKMYGDQHSK ITEEDIK**SGL EGLTVAEALN QK**RLYILDHH<br>**451** DALMPYLRKI NSTKTKTYAT RTLLLLKNDG TLKPLVIELS LPHPQGDQFG<br>**501** ANSKQYFPAE EGVQKSIWQL AKAYVVVNDA GYHQLISHWL NTHAVQEPFV<br>**551** IATHRQLSVL HPIHKLLVPH YKDTMFINAF AR**QVLVNGDG LLEQTHFQSK**<br>**601** YAMELSSYIY KEWNFTEQAL PVDLIKRGVA VEDPSSPNGV KLLIEDYPFA<br>**651** VDGLEIWSTI KTWVTNYCSL YYKDDSAIQN DVELQSWWKE VREKGHVDKK<br>**701** NETWWPKLQN FNELVETCTT IIWISSALHA AVNFGQYPYG GFMPNRPTIS<br>**751** RRLIPAVGSA EYKELESKPE KAYLKTVNSM LQTLLGVSLI EILSRHASDE<br>**801** VYLGQRASIE WTSDKAAVEV FENFGKKVFE VESRIIERNK DVNLKNRSGP<br>**851** VNVPYTLLLP SSTEGLTGRG IPNSISI | | |

atgttttcaattgggaagaatatcattgaaggagctttgaatacaactggggatcttgcgggttctgttatcaatgctggtggtaacattgcagaccaaatctccaatattggaggacagaaaatcaaagggaaa
M·F·S·I·G·K·N·I·I·E·G·A·L·N·T·T·G·D·L·A·G·S·V·I·N·A·G·G·N·I·A·D·Q·I·S·N·I·G·G·Q·K·I·K·G·K

gtgattcttatgagaagtaatgttttggatttcactgaattccattcatctcttcttgataacttcactgagctcttgggtggtggtgtttctcttcaactcattagtgccactcaaacttctgcacttgactca
V·I·L·M·R·S·N·V·L·D·F·T·E·F·H·S·S·L·L·D·N·F·T·E·L·L·G·G·G·V·S·L·Q·L·I·S·A·T·Q·T·S·A·L·D·S

agagggaaagttgggaagaaggcatttctggagagatggctaacgtcaattccaccattgtttgctggagaatcagtgtttcaagtgagctttacatgggaagaaggttttggatttccaggagctatcactatt
R·G·K·V·G·K·K·A·F·L·E·R·W·L·T·S·I·P·P·L·F·A·G·E·S·V·F·Q·V·S·F·T·W·E·E·G·F·G·F·P·G·A·I·T·I

aaaaatggacatacaagtgaattcttccttaaatctctaactcttgaggatgttcctggctttggtcatgtccattttgattgtaattcatgggtttacccatctggaagatacaagaaagatcgtatatttttc
K·N·G·H·T·S·E·F·F·L·K·S·L·T·L·E·D·V·P·G·F·G·H·V·H·F·D·C·N·S·W·V·Y·P·S·G·R·Y·K·K·D·R·I·F·F

gccaataacacatatcttccaagtgatacaccaaatcctcttcgtaagtatagagaggaagaattgttgactcttagaggagatggtactggagagcgtaaggaatgggatagaatatacgactacgatatctac
A·N·N·T·Y·L·P·S·D·T·P·N·P·L·R·K·Y·R·E·E·E·L·L·T·L·R·G·D·G·T·G·E·R·K·E·W·D·R·I·Y·D·Y·D·I·Y

aatgacctttccgaacccggtgatgggcgtcccattcttggaggcagccaatttccttaccctcgtcgtggaagaactggacgaccacgagaatggaaagattccaactatgagagtagattgccagtggtatca
N·D·L·S·E·P·G·D·G·R·P·I·L·G·G·S·Q·F·P·Y·P·R·R·G·R·T·G·R·P·R·E·W·K·D·S·N·Y·E·S·R·L·P·V·V·S

ggattaaacatttacgtaccaagagatgaaaattttggacacttgaagttatcagattttctcggatttgcattgaaatcacttgtagcaacagttcagccagcacttgtaaacatagtagattttacaccagga
G·L·N·I·Y·V·P·R·D·E·N·F·G·H·L·K·L·S·D·F·L·G·F·A·L·K·S·L·V·A·T·V·Q·P·A·L·V·N·I·V·D·F·T·P·G

gagtttgataaattccaagatgttcataatctttatgaaggaggacttcctgttccattggatgtctttagaaatctcaccaaggatttcacacctccaatgttccaagaacttcttaggaccgataatgaccaa
E·F·D·K·F·Q·D·V·H·N·L·Y·E·G·G·L·P·V·P·L·D·V·F·R·N·L·T·K·D·F·T·P·P·M·F·Q·E·L·L·R·T·D·N·D·Q

cgctttctcaaattttcacccccacaagttgttaaagaggacaagtttgcatggcaaacagacgaagaatttgcaagagaaatgctagctggagttaaccctctaatcattcgtcgtcttaaggaatttcctcca
R·F·L·K·F·S·P·P·Q·V·V·K·E·D·K·F·A·W·Q·T·D·E·E·F·A·R·E·M·L·A·G·V·N·P·L·I·I·R·R·L·K·E·F·P·P

aaaagcaaacttgacccaaaaatgtatggtgatcaacatagcaagattaccgaagaagatataaaatctggcttagaaggtcttacagttactgaggcattgaatcagaagagactatacatattggatcaccat
K·S·K·L·D·P·K·M·Y·G·D·Q·H·S·K·I·T·E·E·D·I·K·S·G·L·E·G·L·T·V·T·E·A·L·N·Q·K·R·L·Y·I·L·D·H·H

gatgcattaatgccatatcttagaaaaataaattcaacaaaaacaaaaacatatgccacaagaacattgctacttttgaaaaacgatggaactttgaagccattggttattgagttgagtctgccacaccctcaa
D·A·L·M·P·Y·L·R·K·I·N·S·T·K·T·K·T·Y·A·T·R·T·L·L·L·L·K·N·D·G·T·L·K·P·L·V·I·E·L·S·L·P·H·P·Q

ggtgatcagtttggtgcaaatagcaaacaatattttccagctgaagaaggagttcaaaagtcaatatggcaattggctaaggcttatgtggttgtcaatgatgctggttaccatcaacttatcagccattggttg
G·D·Q·F·G·A·N·S·K·Q·Y·F·P·A·E·E·G·V·Q·K·S·I·W·Q·L·A·K·A·Y·V·V·V·N·D·A·G·Y·H·Q·L·I·S·H·W·L

aatactcatgcagtacaagagccatttgtgattgcgacacatagacaattgagtgtgcttcatccaatccataagttacttgttcctcattacaaagacactatgtttatcaatgcatttgcaagacaagttctt
N·T·H·A·V·Q·E·P·F·V·I·A·T·H·R·Q·L·S·V·L·H·P·I·H·K·L·L·V·P·H·Y·K·D·T·M·F·I·N·A·F·A·R·Q·V·L

gttaatggtgatggtcttcttgaacaaacccactttcaatcaaagtatgccatggagttgtcttcttacatatataaagaatggaatttcactgagcaagcactccctgttgatctcatcaagagaggtgtggcg
V·N·G·D·G·L·L·E·Q·T·H·F·Q·S·K·Y·A·M·E·L·S·S·Y·I·Y·K·E·W·N·F·T·E·Q·A·L·P·V·D·L·I·K·R·G·V·A

gttgaggatccaagttcaccaaatggagttaagttactcatagaggattatccatttgctgtggatgggcttgagatttggtcaacaatcaaaacatgggtcacaaactattgctctctatactacaaagatgat
V·E·D·P·S·S·P·N·G·V·K·L·L·I·E·D·Y·P·F·A·V·D·G·L·E·I·W·S·T·I·K·T·W·V·T·N·Y·C·S·L·Y·Y·K·D·D

agtgcaattcagaatgatgttgaacttcaatcttggtggaaagaggttagagagaaaggtcatgttgataagaaaaatgaaacatggtggccaaagttgcaaaatttcaaggaattggtcgaaacatgtactacc
S·A·I·Q·N·D·V·E·L·Q·S·W·W·K·E·V·R·E·K·G·H·V·D·K·K·N·E·T·W·W·P·K·L·Q·N·F·K·E·L·V·E·T·C·T·T

atcatatggatatcttcggctcttcatgccgcagttaactttggacaatatccttatggtggctttatgccgaatcgaccaacaataagtcgtaggcttatacctgaagtaggaagtgctgagtacaaagaactt
I·I·W·I·S·S·A·L·H·A·A·V·N·F·G·Q·Y·P·Y·G·G·F·M·P·N·R·P·T·I·S·R·R·L·I·P·E·V·G·S·A·E·Y·K·E·L

gaatcaaagcctgagaaggcttacttgaaaacagtcaattcaatgttgcaaacacttcttggagtttcactaattgaaatattatcaaggcatgcttctgatgaagtttatcttggacaaagagctagcattgaa
E·S·K·P·E·K·A·Y·L·K·T·V·N·S·M·L·Q·T·L·L·G·V·S·L·I·E·I·L·S·R·H·A·S·D·E·V·Y·L·G·Q·R·A·S·I·E

tggacttcagacaaagccgccgtggaagtgtttgagaattttggaaaaaaagtgtttgaagttgaaagtagaatcattgaaaggaacaaagatgtgaatctcaagaatagaagtggacctgttaatgttccatat
W·T·S·D·K·A·A·V·E·V·F·E·N·F·G·K·K·V·F·E·V·E·S·R·I·I·E·R·N·K·D·V·N·L·K·N·R·S·G·P·V·N·V·P·Y

actttgcttcttccatcaagtactgaagggctcacaggtagaggaattcctaacagcatctctatt
T·L·L·L·P·S·S·T·E·G·L·T·G·R·G·I·P·N·S·I·S·I

Supplementary Figure S1. Nucleotide and deduced amino acid sequences of *C. sativus* lipoxygenase (CsLOX3). The PLAT and lipoxygenase domains determined using the Pfam server (http://pfam-legacy.xfam.org/) are underlined with green and black lines, respectively. Conserved amino acid residues associated with the binding of iron atoms are indicated in red on a yellow background. Gray color indicates amino acid residues presumably involved in the catalysis of lipoxygenases.

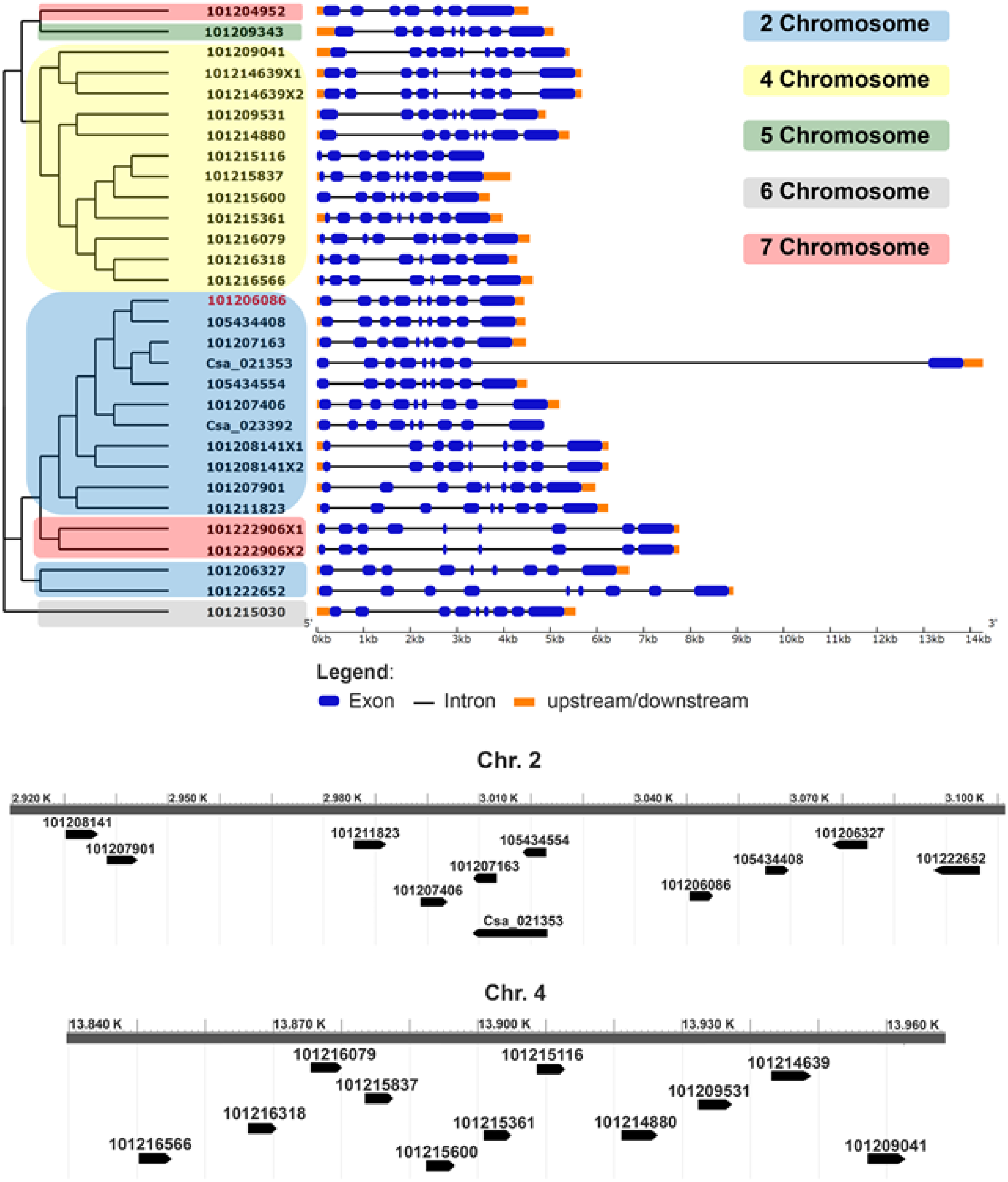


Supplementary Figure S2. Molecular phylogenetic relationship and exon-intron structure of cucumber LOX genes. The numbers are taken from the database and denote the GenBank GeneID. A) Graphic representation of the gene models of thirty LOXs detected in the cucumber genome. A varied number of introns is shown. Exons are shown as blue boxes, and introns are shown as black lines, and orange rectangles represent untranslated regions (UTRs). The lengths of the exon and intron (bp) are shown in kb on the x-axis. B) Two clusters of LOX genes located on chromosomes 2 and 4. The sequence of the gene cloned by us (GI: 101206086) is marked in red.
